# Mesoporous Silica Nanoparticles with Co-Condensed Gadolinium Chelates for Multimodal Imaging

**DOI:** 10.3390/nano2010001

**Published:** 2011-12-27

**Authors:** Kathryn M. L. Taylor-Pashow, Joseph Della Rocca, Wenbin Lin

**Affiliations:** 1 Savannah River National Laboratory, Savannah River Site, Bldg. 773-A, Aiken, SC 29808, USA; Email: kathryn.taylor-pashow@srnl.doe.gov; 2 Department of Chemistry, CB #3290, University of North Carolina at Chapel Hill, Chapel Hill, NC 27599, USA; Email: dellaroc@ad.unc.edu (J.D.R); Email: wlin@email.unc.edu (W.L)

**Keywords:** mesoporous silica nanoparticle, MRI contrast agent, Gd^3+^ contrast agent, multimodal imaging

## Abstract

Several mesoporous silica nanoparticle (MSN) contrast agents have been synthesized using a co-condensation method to incorporate two different Gd^3+^ complexes at very high loadings (15.5–28.8 wt %). These MSN contrast agents, with an MCM-41 type pore structure, were characterized using a variety of methods including SEM and TEM, nitrogen adsorption measurements, thermogravimetric analysis (TGA), direct current plasma (DCP) spectroscopy, and powder X-ray diffraction (PXRD). The magnetic resonance (MR) relaxivities of these contrast agents were determined using a 3 T MR scanner. The *r*_1_ relaxivities of these nanoparticles range from 4.1 to 8.4 mM^−1^s^−1^ on a per Gd basis. Additionally, the MSN particles were functionalized with an organic fluorophore and cancer cell targeting peptide to allow for demonstration of both the optical and MR contrast enhancing capabilities *in vitro*.

## 1. Introduction

Contrast-enhanced magnetic resonance imaging (MRI) is a noninvasive diagnostic technique capable of providing high resolution anatomical images of soft tissue as well as giving quantitative assessment of disease pathogenesis [1,2,3]. Currently used MRI contrast agents are either small molecule gadolinium(III) chelates or simple manganese(II) molecules that are administered in high doses. Small molecule MRI contrast agents currently used in the clinic often cannot provide sufficient image contrast enhancement in early disease stages owing to their lack of sensitivity. Nanoparticle-based MR contrast agents are much more sensitive owing to the enhanced relaxivity on per magnetic center basis as a result of reduced tumbling rates and large payloads of active magnetic centers. Superparamagnetic iron oxide nanoparticles have for example been used as efficient *T*_2_ contrast agents to image tumor angiogenesis, inflammation, and gene expression [4,5,6]. Gd^3+^-containing microemulsions and other lipid particles have on the other hand been shown to be efficient *T*_1_-contrast agents for *in vivo* MR imaging [7]. Several other Gd^3+^-containing solid nanoparticles have also been recently evaluated as potential MRI contrast agents [8,9,10,11,12,13,14,15,16,17,18,19].

We have previously developed a solid silica nanoparticle based MRI contrast agent, which consists of 37 nm particles coated with either a monolayer coating or a polymeric multilayer coating of Gd^3+^ chelates [11]. The results from these two systems revealed that the particles with a multilayer coating had reduced efficiency, or lower relaxivities, on a per Gd^3+^ basis. This was due to the fact that the Gd chelates on the inner layers were not as accessible to the surrounding water molecules [11]. The need for the magnetic centers to be highly accessible to water molecules prompted us to develop new strategies for synthesizing highly efficient nanoparticulate *T*_1_ contrast agents. In order to increase the number of metal centers per particle without reducing the water accessibility of the metal centers, we developed an alternative method for forming the nanoparticulate contrast agents via a layer-by-layer electrostatic self-assembly process [12]. Up to 7 bilayers of alternating cationic Gd(III)-DOTA oligomer and negatively charged polystyrenesulfonate were assembled onto solid silica nanoparticles containing a monolayer of negatively charged Gd chelates. The MR relaxivity on a per Gd basis remained constant, independent of the number of layers assembled. As a result, the relaxivity on a per particle basis increased linearly with the increasing number of layers deposited. We attributed the enhanced MR relaxivity to the highly disordered structure of the layer-by-layer assembly which allows for efficient interaction of all the Gd(III) centers with the surrounding water.

Porous nanostructures represent an alternative platform for incorporating a large number of Gd chelates while keeping them all accessible to the surrounding water. Mesoporous silica materials exhibit high surface areas and tunable pore structures and therefore provide an ideal platform for the development of MR-enhancing hybrid materials. MCM-41 type materials, for example, possess a hexagonal array of one dimensional channels with diameters that can be tuned from 2 nm to 10 nm [20,21]. More recently, synthetic procedures have been developed for controlling the morphologies of MCM-41 materials [22,23], leading to mesoporous silica nanospheres (MSNs) with diameters ranging from 20 to 1,100 nm [24,25,26]. Such hybrid nanomaterials have already been demonstrated in a variety of applications including catalysis [27,28,29,30] and drug delivery [31,32,33,34,35,36,37,38,39]. We recently designed a highly efficient nanoparticulate MR contrast agent by grafting Gd chelates onto MSNs and demonstrated their utility in MR imaging [40]. The loading of Gd chelates in the previously reported MSN system is, however, limited by the grafting efficiency.

In order to further increase the payload of Gd chelates on MSNs, we expored an alternative synthetic strategy based on a co-condesation procedure that was previously reported to be capable of incorporating various organic functional groups into the pores of MSNs [41,42]. In this paper we report the synthesis of mesoporous silica nanoparticles with co-condensed Gd(III) chelates and their applications as optical and MR contrast agents *in vitro*. Two silyl-derived Gd complexes were directly incorporated during the nanoparticle synthesis at different loadings, and the MR contrast enhancing abilities were evaluated. The co-condensation procedure affords MSNs with much higher loadings of Gd(III) chelates, but the *r*_1_ relaxivities of these nanoparticles appear to be smaller than previously reported MSNs with grafted Gd(III) chelates on a per Gd basis, presumably owing to the reduced accessibility of the Gd(III) chelates to the water molecules. A silyl-derived organic fluorophore and targeting agent were also grafted to the surface to allow for target-specific optical and MR imaging of cancer cells.

## 2. Results and Discussion

### 2.1. Synthesis

Two diethylenetriamine pentaacetic acid (DTPA) based ligands were synthesized in this work. The first ligand, 3-aminopropyl(trimethoxysilyl)-diethylenetriamine tetraacetic acid (Si-DTTA), was synthesized by reacting 3-(trimethoxysilylpropyl)diethylene triamine with 4 equivalents of bromoacetic acid under basic conditions. This formed a Gd chelating ligand containing one trialkoxy silane functional group for attachment to the silica based particles. The second ligand, bis(3-aminopropyl triethoxysilyl)-diethylenetriamine pentaacetic acid (Si_2_-DTPA) was synthesized by reacting 2 equivalents of 3-aminopropyltriethoxysilane with DTPA dianhydride in anhydrous pyridine. This provided a Gd chelating ligand with two trialkoxysilane functional groups to allow for incorporation into the mesoporous silica. The corresponding Gd(III) complexes for each ligand were formed by reacting 1 equivalent of GdCl_3_ with the deprotonated form of the ligand. The Gd(III) complexes of Si-DTTA and Si_2_-DTPA are denoted **Gd-1** and **Gd-2**, respectively.

**Scheme 1:**
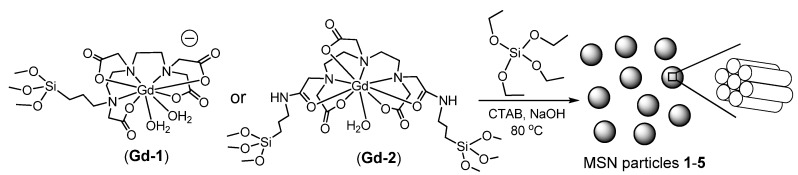
Surfactant template synthesis of mesoporous silica nanoparticles (MSN) **1–5**.

Several MSN-Gd contrast agents were synthesized by incorporating varying amounts of the Si-DTTA-Gd (**Gd-1**) or Si_2_-DTPA-Gd (**Gd-2**) complexes into the particles using a co-condensation method (Scheme 1). Particles were synthesized using 10 wt % **Gd-1** (**1**) and 10, 20, 30, and 40 wt % **Gd-2** complexes (**2–5**) (relative to the amount of TEOS added), using a base-catalyzed condensation reaction. For example, particles of **2**(with 10 wt % **Gd-2** in the feed) were synthesized by simultaneously adding TEOS (0.93 g, 4.48 mmol) and **Gd-2** (0.130 g, 0.134 mmol) to a mixture of CTAB (0.20 g, 0.55 mmol) and 0.7 mL of 2 M NaOH (1.40 mmol) in 240 mL of water at 80 °C. The reaction mixture was then stirred at 80 °C for 2 h. The particles were isolated by centrifuging and washed with water and ethanol. The surfactant template was then extracted with a 1 wt % solution of NaCl in methanol at room temperature. In order to ensure the removal of any free Gd^3+^ ions, the particles were also washed with a pH = 3 solution.

### 2.2. Characterization

**Figure 1:**
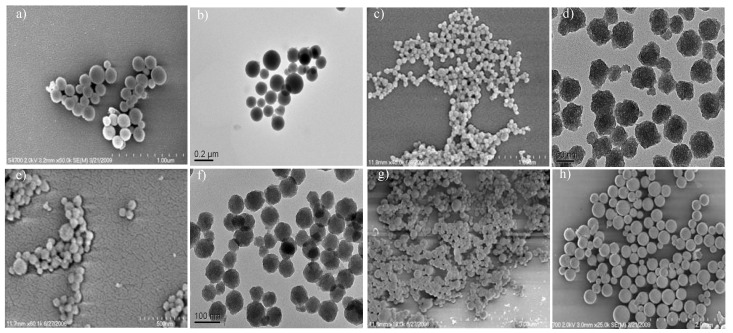
SEM and TEM images of MSN samples **1 **(a,b), **2** (c,d), and **3** (e,f). SEM images of MSN samples **4** (g) and **5 **(h).

Scanning electron and transmission electron microscopies (SEM and TEM) were used to evaluate the particle size and morphology. The particles ranged in size from approximately 75 nm to several hundred nanometers, with the size generally increasing with increasing wt % of the Gd complex (Figure 1). The SEM and TEM images also showed some evidence of slight aggregation and fusing.

**Figure 2:**
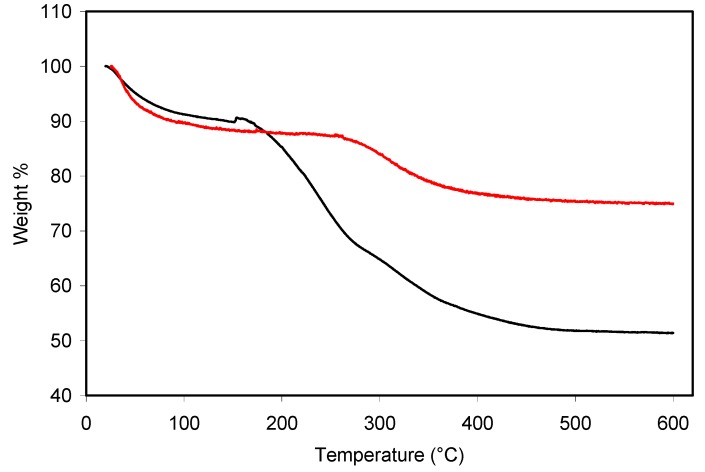
TGA of **2** before (black) and after (red) surfactant extraction.

TGA was used to characterize the amount of Gd chelate that had been incorporated in each of the products from the co-condensation reactions (Figure 2 and Figures S1–S4). After extraction of the surfactant, the weight loss in the 230–450 °C temperature range corresponds to the organic portion of the incorporated Gd complexes. The precise amount of Gd complex that had been incorporated was then determined by measuring the [Gd] of a digested particle solution using direct current plasma (DCP) spectroscopy. The TGA and DCP gave fairly consistent results as to the amount of incorporated Gd complex (Table 1). The amount of the incorporated Gd(III) chelates tracks fairly well with that in the feed, but appears to reach a maximum loading at ~30 wt %.

**Table 1:** Determination of incorporated Gd(III) complexes (wt %) in MSNs by TGA and DCP.

Particles	Gd complex in the feed (wt %)	Incorporated Gd complex by TGA	Gd content by DCP	Incorporated Gd complex by DCP
1	Gd-1 (10)	16.0	5.3	15.2
2	Gd-2 (10)	15.5	3.8	15.1
3	Gd-2 (20)	23.1	4.7	18.7
4	Gd-2 (30)	27.7	6.8	26.8
5	Gd-2 (40)	28.8	8.1	32

**Figure 3:**
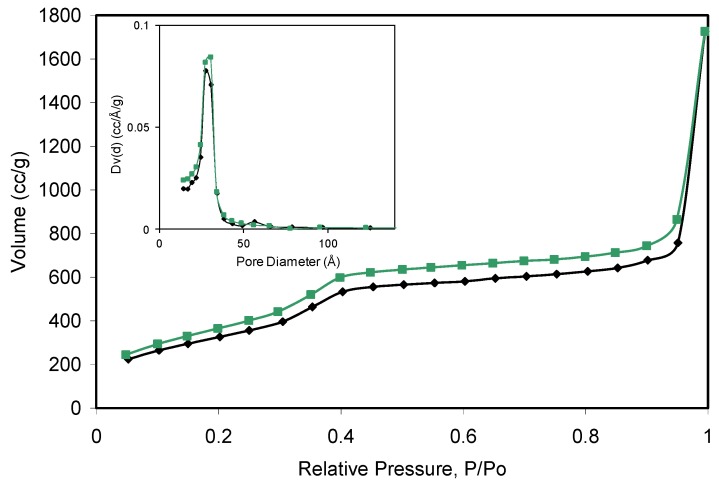
Nitrogen sorption isotherm for **2**. The inset shows the pore size distribution. Black color represents adsorption and green color represents desorption data.

The pore structure of the particles was evaluated using powder X-ray diffraction (PXRD) and nitrogen gas sorption measurements. Pristine MCM-41 particles synthesized in our lab exhibit PXRD peaks at 2θ values of 2.8°, 4.6°, and 5.4° that are characteristic of the (100), (110), and (200) planes of the MCM-41 material, respectively. PXRD of the co-condensed samples shows a similar pattern, although the peak is much broader indicating less defined pore structure (Figure S5). Nitrogen gas sorption measurements indicated that the surfactant-extracted MSN particles are highly porous with BJH (Barrett-Joiner-Halenda) surface areas ranging from 149 to 1674 m^2^/g and have pore diameters ranging from 1.4 to 2.9 nm (Figure 3 and Figures S6–S13). The surface areas and pore sizes decrease with increasing incorporation of **Gd-2** (Table 2).

**Table 2:** Summary of Barrett-Joiner-Halenda (BJH) surface areas and pore sizes.

Particle	Gd complex in the Feed (wt %)	Surface area (m^2^/g)	Pore size (nm)
1	Gd-1 (10)	832	1.94
2	Gd-2 (10)	1674	2.91
3	Gd-2 (20)	1095	2.75
4	Gd-2 (30)	923	2.46
5	Gd-2 (40)	149	1.41

**Figure 4:**
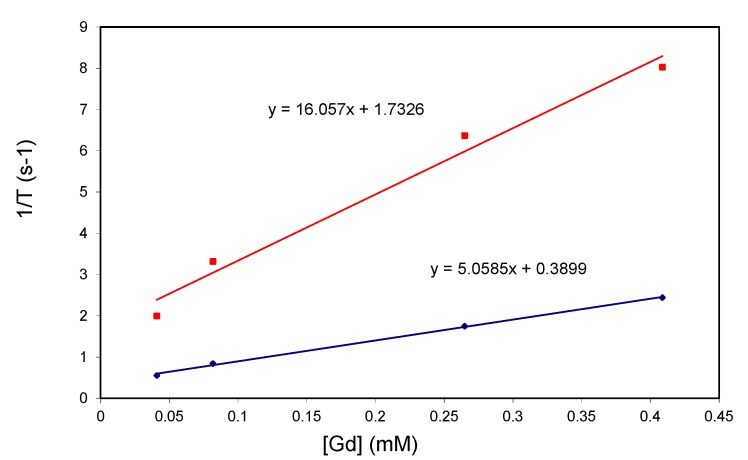
*r*_1_ (blue) and *r*_2_ (red) relaxivity curves of **2** measured at 3 T.

**Table 3:** Summary of relaxivities measured at 3 T.

Particles	*r_1_*(mM^−1^s^−1^)	*r_2_* (mM^−1^s^−1^)
1	6.2	32.7
2	5.1	16.1
3	4.1	25.3
4	4.8	22.9
5	8.4	25.6

### 2.3. In Vitro Experiments

For *in vitro* experiments, particles **4** were tagged with an organic fluorophore to enable visualization of the particles using confocal microscopy. The particles were also made target specific by grafting an RGD peptide onto the surface. This peptide sequence targets the α_v_β_3_ integrin, which is over expressed on many types of cancer cells [43].

**Figure 5:**
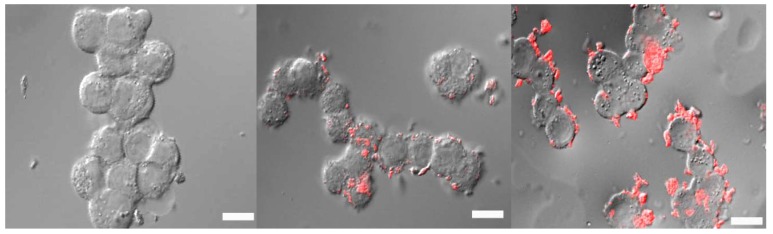
Overlaid DIC and Fluorescence Image of HT-29 colon cancer cells incubated with no MSN (left), 500 μg MSN (center), or 500 μg MSN-RGD (**4a**) (right). All scale bars indicate 25 μm.

Laser scanning confocal fluorescence microscopy images indicated the localization of the nanoparticles on the surface of the HT-29 human colorectal adenocarcinoma cells after 30 min of incubation (Figure 5 and Figure S18). The cells incubated with no particle (Figure 5, left) showed no rhodamine fluorescence, while the cells incubated with particles showed significant fluorescence. Addition of the cRGD peptide did not appear to induce internalization of the nanoparticles through receptor mediated endocytosis, but did increase localization of the particles on the cell surface.

**Figure 6:**
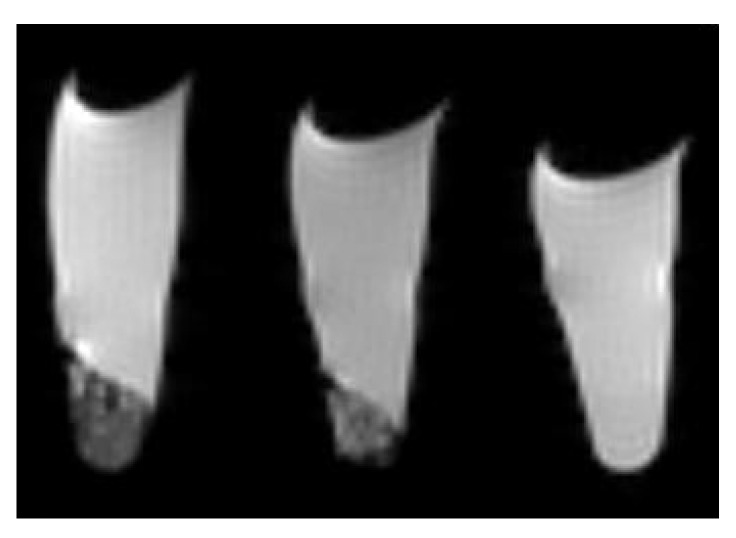
T_2_ Weighted MRI image (9.4T) of HT-29 cells incubated with no MSN (right), 300 µg MSN (**4**) (center), and 300 µg MSN-RGD (**4a**) (left).

MRI imaging on a 9.4T scanner showed that the nanoparticles gave *T*_2_-weighted enhancement of HT-29 cells incubated with nanoparticles **4** for 1 h (Figure 6). The pronounced *T*_2_-weighted enhancement is expected given the much higher *r*_2_/*r*_1_ ratios for these MSN particles than the previously reported MSNs with post-synthetically grafted Gd(III) chelates [40]. Cells incubated with nanoparticles showed increased *T*_2_-weighted contrast compared to the control. In addition, cells incubated with the targeted nanoparticles displayed enhanced *T*_2_-weighted contrast compared to cells incubated with the non-targeted nanoparticles. The *in vivo* utility of the present co-condensed MSN nanoparticles is, however, limited due to their relatively large sizes and non-degradable nature. The particles cannot be cleared from the kidney, and as the particles stay in the organs for an extended period of time, the leaching of toxic Gd^3+^ ions from the particles becomes a significant concern.

## 3. Experimental Section

Cetyltrimethylammonium bromide (CTAB), GdCl_3_∙6H_2_O, bromoacetic acid, and tetraethyl orthosilicate (TEOS) were purchased from Aldrich and used without further purification. 3-(trimethoxysilylpropyl)diethylene triamine, (3-isocyanatopropyl)triethoxysilane, and 3-aminopropyltriethoxysilane were purchased from Gelest. All other chemicals were purchased from Fisher Scientific and used without further purification. Thermogravimetric analysis (TGA) was performed under air using a Shimadzu TGA-50 equipped with a platinum pan at a heating rate of 3 °C per minute. Powder X-ray diffraction (PXRD) patterns were collected on a Bruker SMART APEX II diffractometer using Cu radiation. The PXRD patterns were processed with the APEX 2 package using the phase ID plug-in. A Hitachi 4700 field emission scanning electron microscope (SEM) and a JEM 100CX-II transmission electron microscope (TEM) were used to determine particle size and morphology. A Cressington 108 Auto Sputter Coater equipped with a Au/Pd (80/20) target and an MTM-10 thickness monitor was used to coat the samples with a 5 nm thick conductive layer before taking SEM images. Each SEM sample was prepared by suspending the nanoparticles in ethanol. A drop of the suspension was then placed on a glass slide and the solvent was allowed to evaporate. TEM samples were also prepared from ethanolic particle dispersions on amorphous carbon coated copper grids. An Applied Research Laboratories (ARL) SpectraSpan 7 DCP spectrometer was used to measure Gd^3+^ concentrations.

**Synthesis of 3-aminopropyl(trimethoxysilyl)-diethylenetriamine tetraacetic acid (Si-DTTA)**. Bromoacetic acid (0.5558 g, 4.00 mmol) and 3-(trimethoxysilylpropyl)-diethylene triamine (0.2654 g, 1.00 mmol) were dissolved in 1.0 mL of distilled water and 2.0 mL 2 M sodium hydroxide (4.00 mmol) with magnetic stirring. The reaction mixture was subsequently heated to 50 °C, and an additional 3.0 mL of 2 M NaOH was added dropwise over approximately 30 min. After stirring for an additional 2 h at 50 °C, the solvent was removed under reduced pressure to yield a viscous yellow oil. An off-white hygroscopic powder was isolated from the oil in high yield (>90%) by precipitation with ethanol and subsequent drying under vacuum. MS (ESI negative ion): *m/z *542.2 [M-H]^−^ for the silanetriol from a basic solution. NMR: ^1^H (D_2_O, 300 MHz): δ 0.47 (2H), 1.55 (2H), 2.62–2.78 (10H), 3.14–3.21 (8H).

**Synthesis of Si-DTTA-Gd Complex (Gd-1)**. The gadolinium complex was prepared by dissolving the isolated Si-DTTA product (108.6 mg, 0.2 mmol) in 4 mL of distilled water with magnetic stirring at room temperature. GdCl_3_ (380 μL of a 0.50 M solution, 0.19 mmol) was slowly titrated into the solution while maintaining a pH of ~9 with the dropwise addition of 2 M NaOH. The reaction was then stirred at room temperature for an additional 2 h. The resultant solution was used directly.

**Synthesis of Bis(3-aminopropyl triethoxysilyl)-diethylenetriamine pentaacetic acid ( Si_2_-DTPA) **. Diethylenetriamine pentaacetic acid dianhydride (5.000 g, 13.995 mmol) was dissolved in 110 mL of anhydrous pyridine under a steady flow of nitrogen. Using standard Schlenk line techniques 3-aminopropyl triethoxysilane (6.85 g, 31.00 mmol) was added and the resultant reaction mixture was magnetically stirred under nitrogen for 24 h. The product was then precipitated with copious amounts of hexane, isolated via centrifuging, washed with additional aliquots of hexanes, and dried to yield 10.436 g (93.2 %) of the desired compound (Si_2_-DTPA). MS (ESI negative ion): *m/z *631.3 [M-H]^−^ for the silanetriol from a basic solution. NMR: ^1^H (DMSO-d_6_): δ 0.52 (t, 4H), 1.14 (t, 18H), 1.44(p, 4H), 2.81 (t, 4H), 2.92 (t, 4H), 3.04 (q, 4H), 3.22 (s, 6H), 3.34 (s, 4H), 3.73 (q, 12H), 8.06 (t, 2H). ^13^C{1H} (DMSO-d_6_): δ 8.0, 18.8, 23.4, 41.8, 51.2, 52.8, 55.9, 56.7, 58.3, 58.4, 170.7, 173.4.

**Synthesis of Si_2_-DTPA-Gd Complex (Gd-2)**. To prepare the gadolinium complex, Si_2_-DTPA (1.77 g, 2.22 mmol) was dissolved in ~3 equivalents of NaOH (6.0 mL of a 1.0 M solution) with magnetic stirring for 30 min. To this solution was added 0.90 equivalent of GdCl_3_ (4.0 mL of a 0.5 M solution, 0.002 mol) and the mixture was magnetically stirred at room temperature for several hours, the volume of the solution was adjusted to 10 mL to yield a visibly clear yellow 0.20 M solution of the modified gadodiamide complex.

**Synthesis of co-condensed MSN with 10 wt % Gd-1 in the feed (1)**. 0.04 g (0.11 mmol) of CTAB was dissolved in 48 mL of H_2_O containing 0.14 mL of 2 M NaOH (0.28 mmol). The solution was heated to 80 °C. After reaching 80 °C, 0.2 mL of TEOS, and 2.54 mL (0.036 mmol) of a 0.014 M aqueous solution of **Gd-1** were then added. The reaction was then stirred for an additional 2 h at 80 °C. The product was isolated by centrifuging, and washed with water, acidic water (pH = 3), and ethanol. The surfactant was extracted (3 times) using 1 wt % NaCl in methanol. Yield: 32.9 mg.

**Synthesis of co-condensed MSN with 10 wt % Gd-2 in the feed (2)**. 0.20 g (0.55 mmol) of CTAB was dissolved in 240 mL of H_2_O containing 0.70 mL of 2 M NaOH (1.4 mmol). The solution was heated to 80 °C. After reaching 80 °C, 1.0 mL of TEOS, and 0.79 mL (0.134 mmol) of a 0.17 M aqueous solution of **Gd-2** were then added. The reaction was then stirred for an additional 2 h at 80 °C. The product was isolated by centrifuging, and washed with water, acidic water (pH = 3), and ethanol. The surfactant was extracted using the same procedure as above. Yield: 150.1 mg.

**Synthesis of co-condensed MSN with 20 wt % Gd-2 in the feed (3)**. 0.20 g (0.549 mmol) of CTAB was dissolved in 240 mL of H_2_O containing 0.70 mL of 2 M NaOH (1.40 mmol). The solution was heated to 80 °C. After reaching 80 °C, 1.0 mL of TEOS, and 1.37 mL (0.233 mmol) of a 0.17 M aqueous solution of **Gd-2** were then added. The reaction was then stirred for an additional 2 h at 80 °C. The product was isolated by centrifuging, and washed with water, acidic water (pH = 3), and ethanol. The surfactant was extracted using the same procedure as above. Yield: 326.1 mg.

**Synthesis of co-condensed MSN with 30 wt % Gd-2 in the feed (4)**. 0.20 g (0.549 mmol) of CTAB was dissolved in 240 mL of H_2_O containing 0.70 mL of 2 M NaOH (1.40 mmol). The solution was heated to 80 °C. After reaching 80 °C, 1.0 mL of TEOS, and 2.04 mL (0.347 mmol) of a 0.17 M aqueous solution of **Gd-2** were then added. The reaction was then stirred for an additional 2 h at 80 °C. The product was isolated by centrifuging, and washed with water, acidic water (pH = 3), and ethanol. The surfactant was extracted using the same procedure as above. Yield: 241.0 mg.

**Synthesis of co-condensed MSN with 40 wt % Gd-2 in the feed (5)**. 0.220 g (0.603 mmol) of CTAB was dissolved in 240 mL of H_2_O containing 0.70 mL of 2 M NaOH (1.40 mmol). The solution was heated to 80 °C. After reaching 80 °C, 1.0 mL of TEOS, and 3.03 mL (0.455 mmol) of a 0.15 M aqueous solution of **Gd-2** were then added. The reaction was then stirred for an additional 2 h at 80 °C. The product was isolated by centrifuging, and washed with water, acidic water (pH = 3), and ethanol. The surfactant was extracted using the same procedure as above. Yield: 276.3 mg.

**Synthesis of Rhodamine-APS**. 6.8 mg (0.0127 mmol) of rhodamine B isothiocyanate was dissolved in 1.1 mL of ethanol. 3.3 µL (3.1 mg, 0.0141 mmol) of 3-aminopropyltriethoxysilane was then added, and the reaction was stirred at room temperature, under N_2_, and in the dark for 24 h. At the completion of the reaction the solution was diluted to a total volume of 2 mL with additional ethanol to make a solution with a rhodamine-APS concentration of approximately 6 mM.

**Synthesis of tri(ethoxy)silylpropyl carbamoyl c(RGDfK)**. Cyclic(RGDfK) (2.0 mg, 3.313 μmol) was placed in a small round bottom flask and dried under high vacuum for 1 h. The c(RGDfK) was then dissolved in 0.5 mL of anhydrous DMSO and 0.2 μL of Hünig base. 0.86 μL (3.44 μmol) of (3-isocyanatopropyl)triethoxysilane was then added, and the reaction was stirred under argon for 18 h. The solution (4 mg c(RGDfK)/mL DMSO) was placed in a freezer for later use.

**Rhodamine B and RGD functionalized MSN (4a)**. 46 mL of ethanol was placed in a round bottom flask, and 1.2 mL of NH_4_OH (3 vol%) was added. 4 mL of a 5 mg/mL suspension of **4** was then added followed by 200 µL of a 4 mg/mL solution of Si-c(RGDfK) and 66 µL of a 6 mM solution of rhodamine-APS. The reaction was then stirred at room temperature, in the dark, for 18 h. The particles were then isolated by centrifuged at 10,000 rpm for 10 min, and were washed with water and ethanol before being redispersed in ethanol.

**Relaxivity Measurements. **MR relaxivities were determined on a Siemens 3 T Allegra (Siemens Medical Systems, Erlangen, Germany) with a CP head coil. A 3D FLASH sequence was utilized to compute *T*_1_ maps with seven different flip angles (2°, 5°, 10°, 20°, 30°, 40°, and 60°). Imaging parameters were: FOV = 190 × 190 × 64 mm^3^, Matrix size = 128 × 128 × 32, TR/TE = 40/1.64 ms, total data acquisition time was 19 min. A 2-D multiple echo spin echo sequence was used to estimate *T*_2_ maps. In total, 32 echoes with an echo spacing of 6.2 ms were obtained. The first echo time was 6.2 ms. TR was 3,000 ms. FOV and matrix size were set to 190×190 mm^2 ^and 128×128. The slice thickness was 2 mm. The total data acquisition time was about 6 min and 29 seconds.

**Confocal microscopic and MRI imaging.** HT-29 colon adenocarcinoma (ATCC Number: HTB-38) were cultured in McCoy’s 5A (Cellgro, Manassas, VA, USA) supplemented with 10% Fetal Bovine Serum (Aldrich, St. Louis, MO, USA), 200IU penicillin, and 200 μg streptomycin (Cellgro). Cells were incubated at 37 °C with 5% CO_2_.

HT-29 cells were collected by trypsinization and were diluted to 300,000 cells/mL with McCoy’s 5A media. One mL of the suspension was placed on a glass coverslip in the center of a six well cell plate. Three mL of cell media was then added around the suspension and the plate was incubated for 24 h. The plate was removed from the incubator and the media was removed, washed once with PBS and replaced with 3 mL fresh media. At this point, 500 μg of nanoparticles (1 mL media) were added. The plate was then incubated for an additional 30 min. The plate was removed, the wells washed twice with PBS and 1 mL fresh media was added. The coverslips were removed from the wells and imaged at the microscopy services laboratory of the Department of Pathology and Laboratory Medicine at UNC-Chapel Hill. An Olympus FV500 Confocal Laser Scanning Microscope was used with DIC settings. The RITC tagged nanoparticles were imaged using 543 nm excitation and a 560 nm long pass emission filter.

HT-29 cells were plated in 25 cm^3^ T-necked flasks at 3.3 million cells/flask in 7 mL media (3 flasks). The flasks were returned to the incubator overnight. The next day, the flasks were removed from the incubator. The media was removed from the flask, washed once with PBS, and 5 mL fresh media was added. Each flask then received either 0.3 mg of non-targeted nanoparticle, 0.3 mg of targeted nanoparticle, or no nanoparticle in 0.5 mL PBS. The flasks were returned to the incubator for 1 h. The media was then removed from each flask, washed thrice with PBS, and 2 mL of trypsin-EDTA was added to each flask to detach the cell monolayers. The flasks were returned to the incubator for 10 min. The cells were then isolated by centrifugation and redispersed into 200 μL PBS. The cells were re-pelleted in PCR tubes and imaged on a 9.4 T scanner at the Small Animal Imaging Facility at UNC-CH.

## 4. Conclusions

We have designed and characterized several hybrid mesoporous silica nanospheres (MSN-Gd) with strong ability to enhance MR images. Two different Gd chelates were incorporated into MSN particles with the MCM-41 type pore structure using a co-condensation method. The present co-condensation procedure affords much higher loadings of Gd(III) chelates, and the MR relaxivities of these nanoparticles on a per Gd basis appear to be larger than the small molecule Gd(III) chelates. The utility of the mesoporous silica nanospheres with co-condensed Gd(III) chelates as contrast agents for optical and MR imaging has been demonstrated *in vitro*.

## Supplementary Materials

Supplementary File 1
