# Supplementary material for: Mesoporous Silica Nanoparticles with Co-Condensed Gadolinium Chelates for Multimodal Imaging
Source: Nanomaterials (Basel). 2011 Dec 27;2(1):1–14. doi: 10.3390/nano2010001 (PMC3920552; doi:10.3390/nano2010001)

Nanomaterials **2012**, *2*, 1-14; doi:10.3390/nano2010001

OPEN ACCESS

nanomaterials

ISSN 2079-4991

www.mdpi.com/journal/nanomaterials

Article

Mesoporous Silica Nanoparticles with Co-Condensed Gadolinium Chelates for Multimodal Imaging

Kathryn M. L. Taylor-Pashow [1,†], Joseph Della Rocca [2,†] and Wenbin Lin [2,*]

[1] Savannah River National Laboratory, Savannah River Site, Bldg. 773-A, Aiken, SC 29808, USA; E-Mail: kathryn.taylor-pashow@srnl.doe.gov

[2] Department of Chemistry, CB #3290, University of North Carolina at Chapel Hill, Chapel Hill, NC 27599, USA; E-Mail: dellaroc@ad.unc.edu

† These authors contribute equally to this work.

* Author to whom correspondence should be addressed; E-Mail: wlin@email.unc.edu; Tel.: +1-919-962-6320; Fax: +1-919-962-2388.

Supplementary Information

Figure S1. TGA of **1** before (black) and after (red) surfactant extraction.

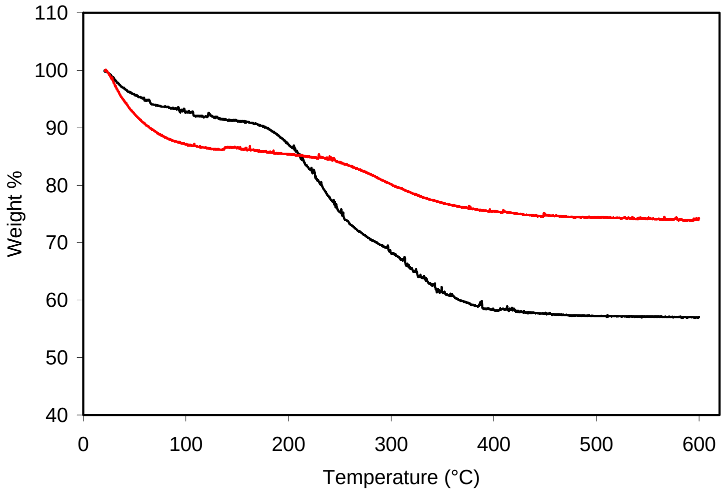

Figure S2. TGA of **3** before (black) and after (red) surfactant extraction.

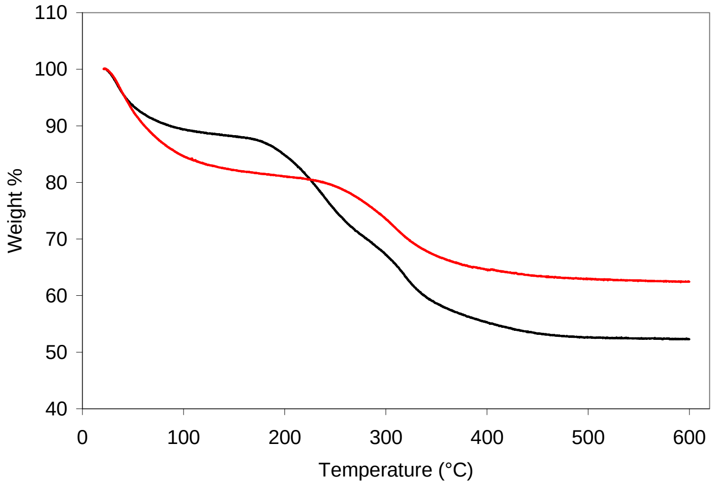

Figure S3. TGA of **4** before (black) and after (red) surfactant extraction.

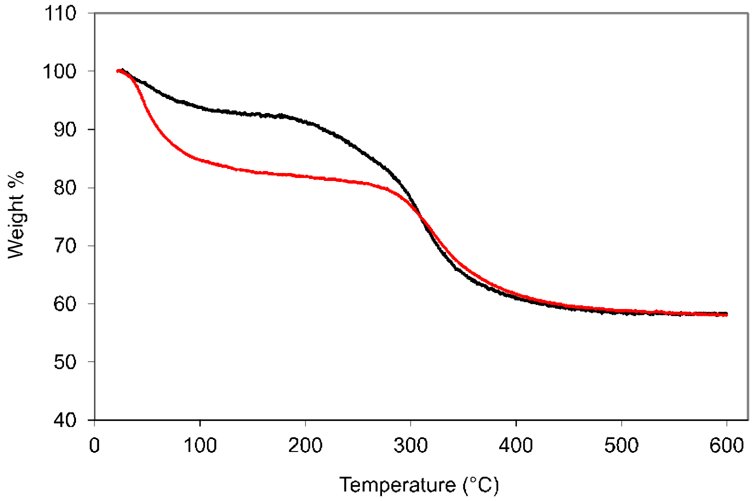

Figure S4. TGA of **5** before (black) and after (red) surfactant extraction.

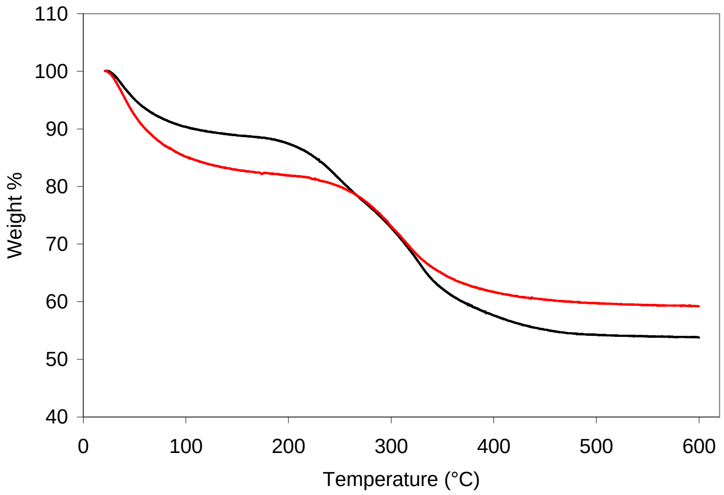

Figure S5. PXRD of MCM-41 nanoparticles (black) and co-condensed samples **1** (red), **2** (blue), **3** (orange), **4** (green), and **5** (pink).

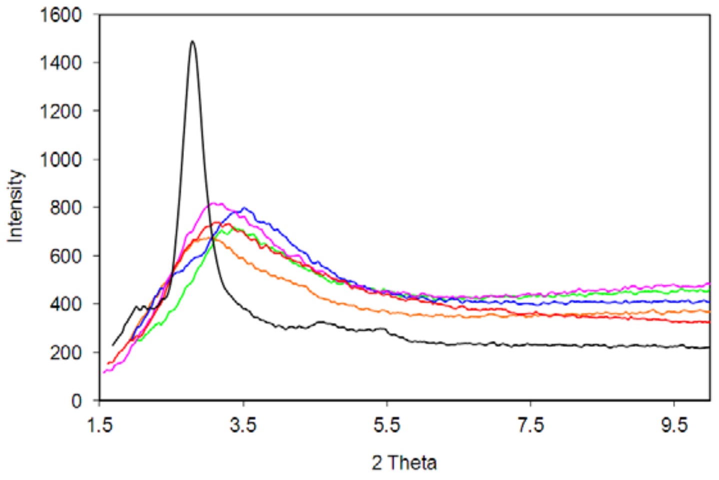

Figure S6. Nitrogen adsorption isotherm of **1** (black-adsorption; green-desorption).

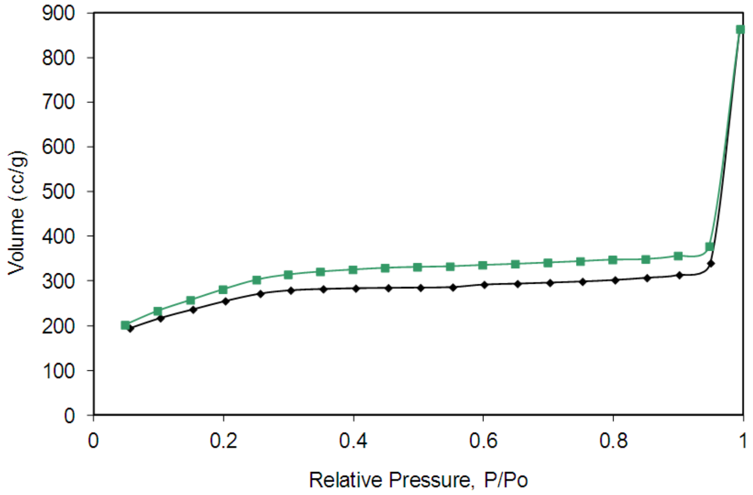

Figure S7. Pore size distribution of **1** (black-adsorption; green-desorption).

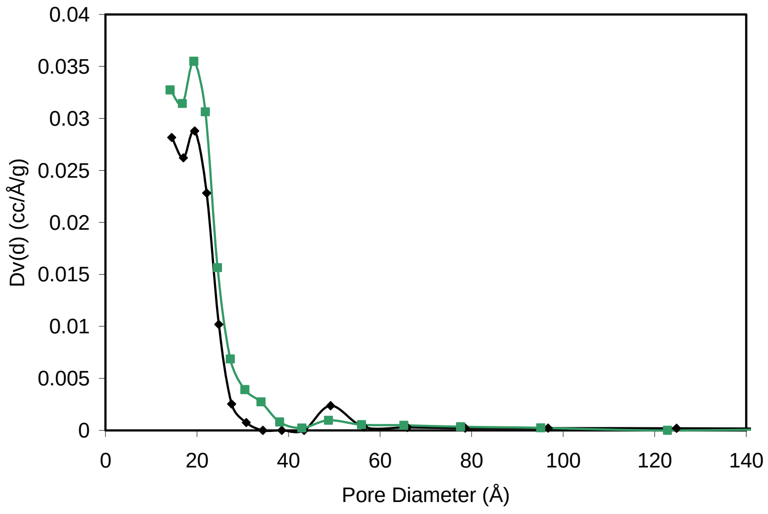

Figure S8. Nitrogen adsorption isotherm of **3** (black-adsorption; green-desorption).

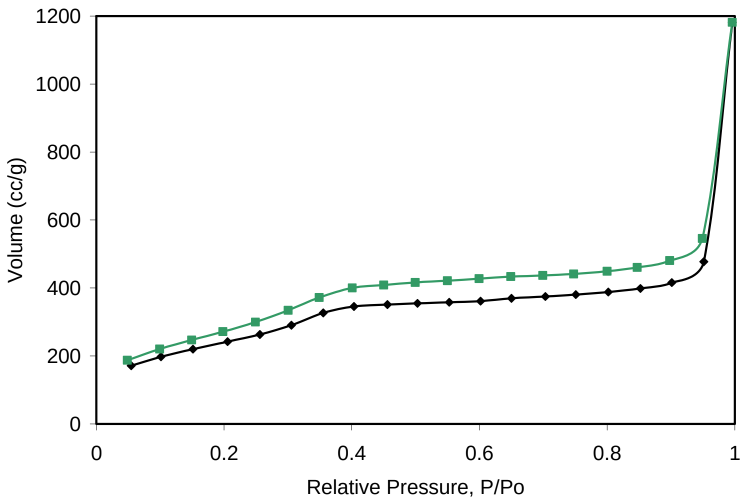

Figure S9. Pore size distribution of **3** (black-adsorption; green-desorption).

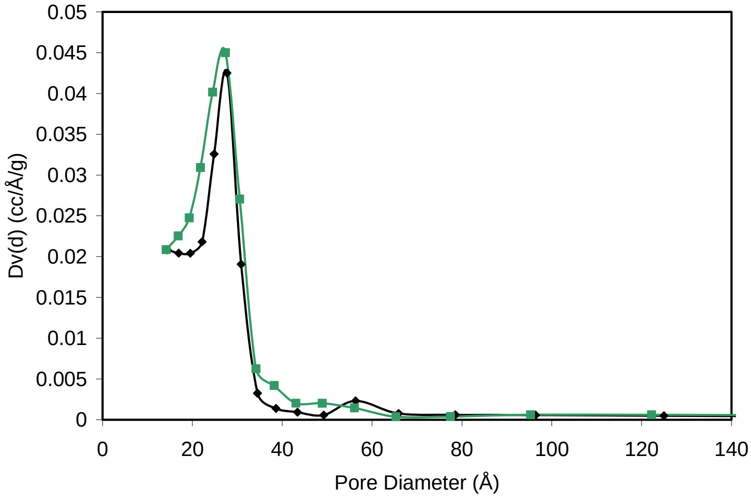

Figure S10. Nitrogen adsorption isotherm of **4** (black-adsorption; green-desorption).

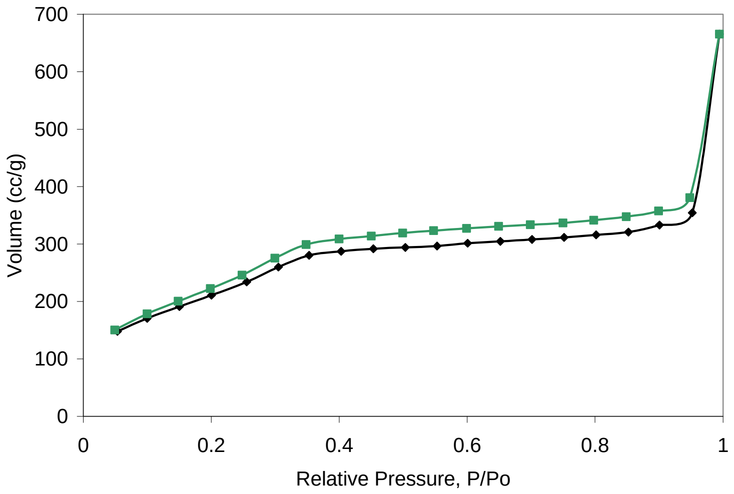

Figure S11. Pore size distribution of **4** (black-adsorption; green-desorption).

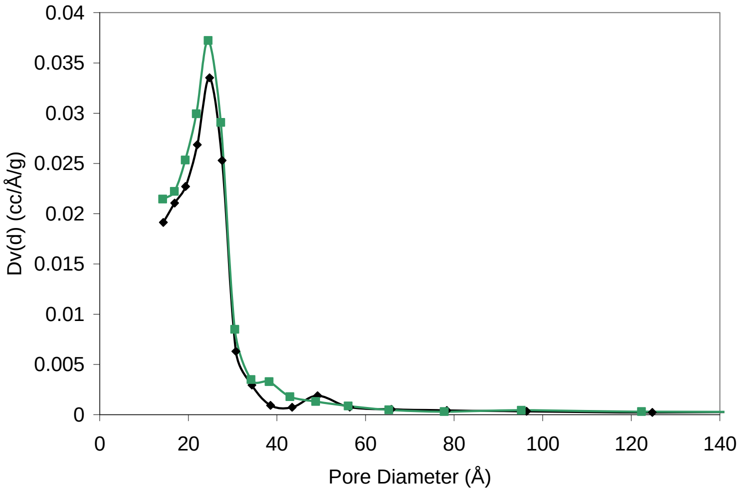

Figure S12. Nitrogen adsorption isotherm of **5** (black-adsorption; green-desorption).

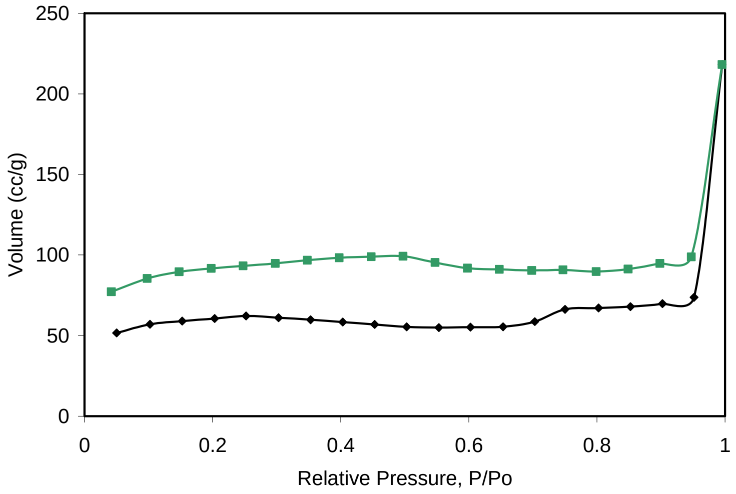

Figure S13. Pore size distribution of **5** (black-adsorption; green-desorption).

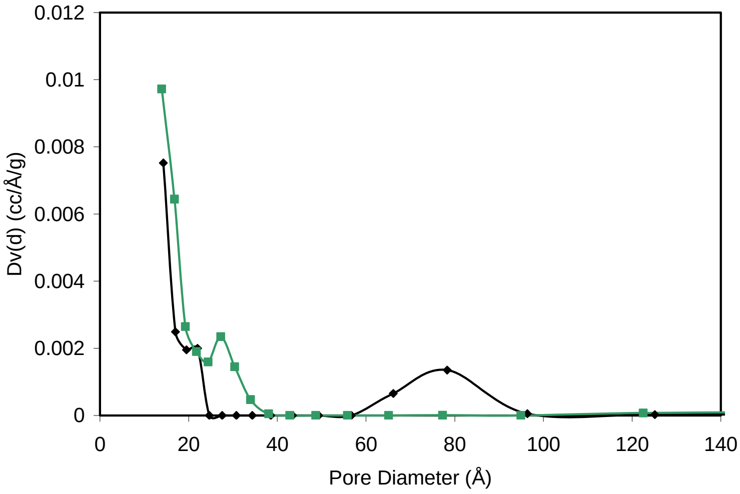

Figure S14. r_1 (blue) and r_2 (red) relaxivity curves of **1** measured at 3 T.

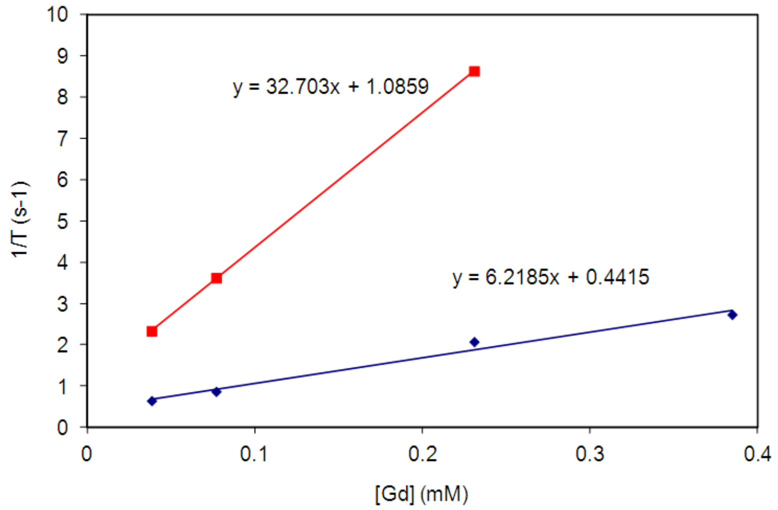

Figure S15. r_1 (blue) and r_2 (red) relaxivity curves of **3** measured at 3 T.

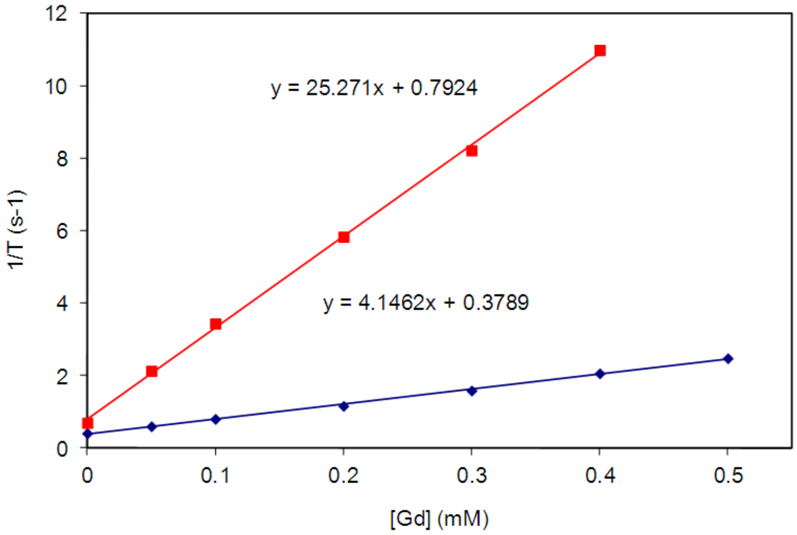

Figure S16. r_1 (blue) and r_2 (red) relaxivity curves of **4** measured at 3 T.

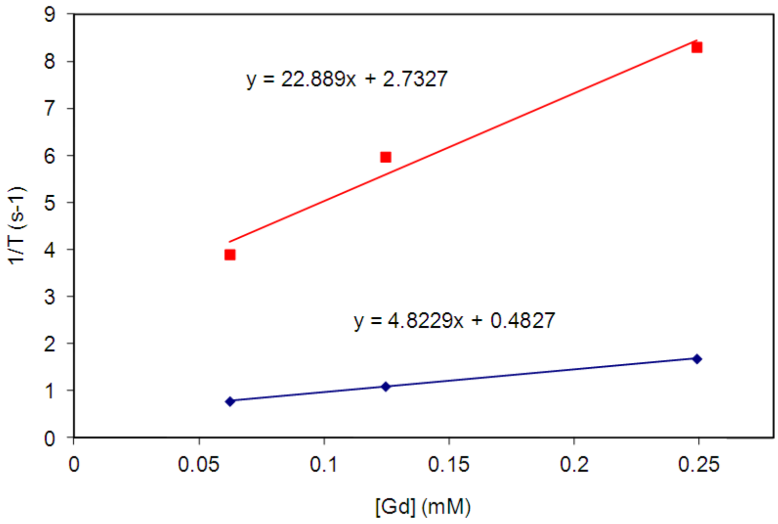

Figure S17. r_1 (blue) and r_2 (red) relaxivity curves of **5** measured at 3 T.

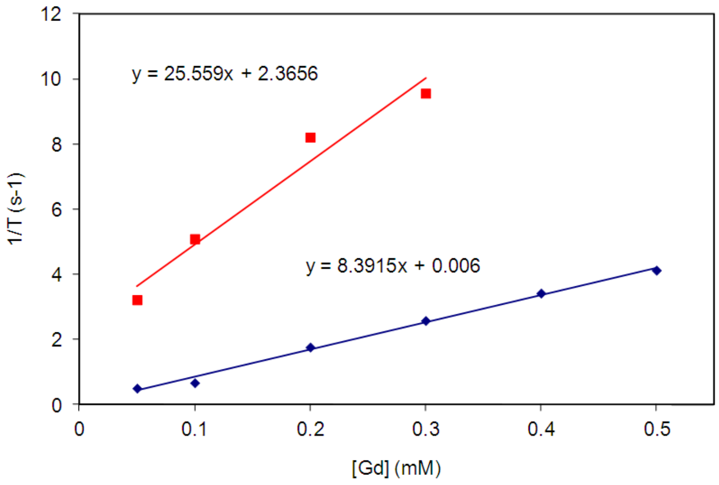

Figure S18. Confocal Scanning Laser Microscopy images of HT-29 cells incubated with no particle (**top**), 500 µg **4** (**middle**), or 500 µg **4**-RGD (**bottom**). The rhodamine fluorescence channel is on the left and the DIC image is on the right. The scale bar designates 25 µm.

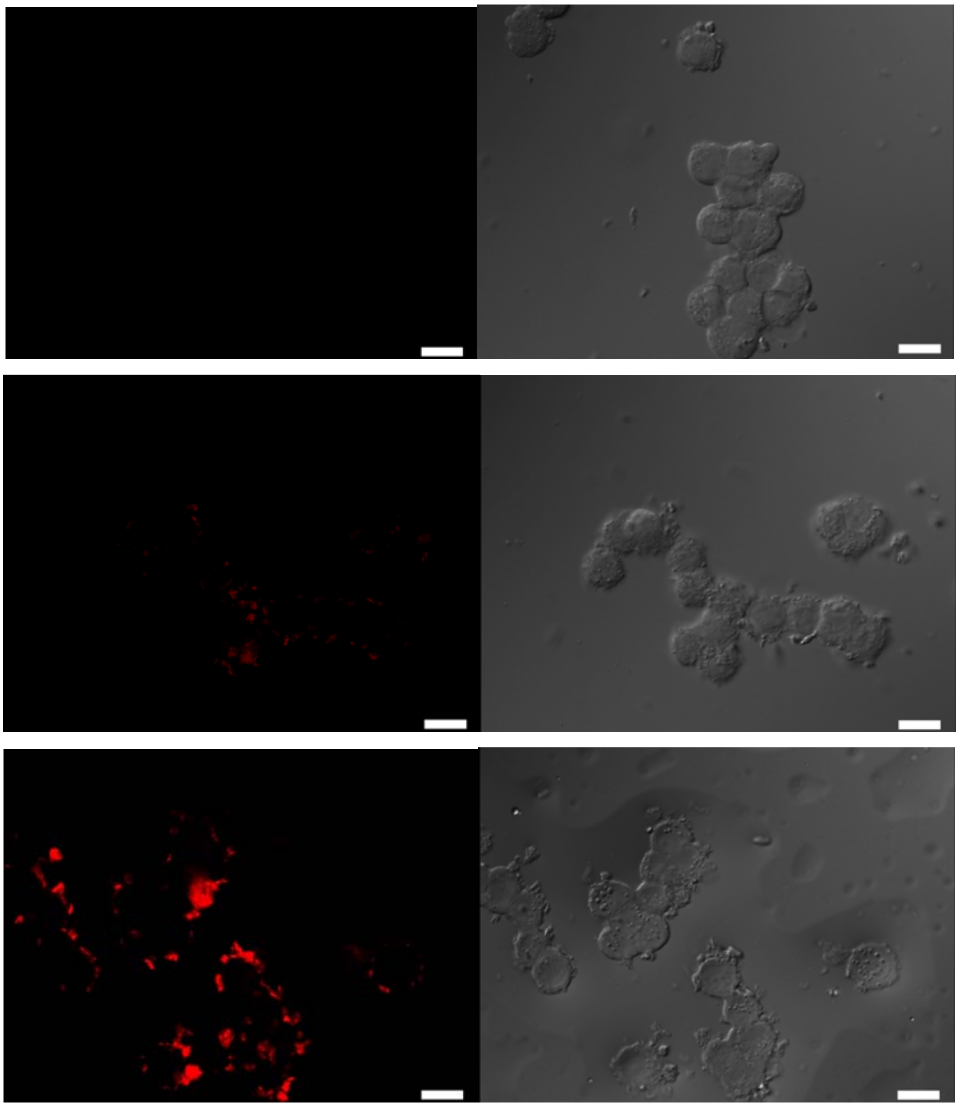

Supplement: Supplementary File 1 [file nanomaterials-02-00001-s001.pdf]
